# Epistatic interaction between *Rhg1-a and Rhg2* in PI 90763 confers resistance to virulent soybean cyst nematode populations

**DOI:** 10.1007/s00122-022-04091-2

**Published:** 2022-04-05

**Authors:** Pawan Basnet, Clinton G. Meinhardt, Mariola Usovsky, Jason D. Gillman, Trupti Joshi, Qijian Song, Brian Diers, Melissa G. Mitchum, Andrew M. Scaboo

**Affiliations:** 1 Division of Plant Science and Technology, University of Missouri, Columbia, MO 65211 USA; 2 Plant Genetics Research Unit, USDA-ARS, Columbia, MO USA; 3 Department of Health Management and Informatics, MUIDSI, and Bond Life Sciences Center, University of Missouri-Columbia, Columbia, MO 65211 USA; 4 Soybean Genomics and Improvement Laboratory, Beltsville Agricultural Research Center, USDA-ARS, Beltsville, MD USA; 5 Department of Crop Sciences, University of Illinois, Urbana-Champaign, IL USA; 6 Department of Plant Pathology and Institute of Plant Breeding, Genetics and Genomics, University of Georgia, Athens, GA USA

## Abstract

**Key message:**

An epistatic interaction between SCN resistance loci *rhg1-a* and *rhg2* in PI 90763 imparts resistance against virulent SCN populations which can be employed to diversify SCN resistance in soybean cultivars.

**Abstract:**

With more than 95% of the $46.1B soybean market dominated by a single type of genetic resistance, breeding for soybean cyst nematode (SCN)-resistant soybean that can effectively combat the widespread increase in virulent SCN populations presents a significant challenge. *Rhg* genes (for Resistance to *Heterodera glycines*) play a key role in resistance to SCN; however, their deployment beyond the use of the *rhg1-b* allele has been limited. In this study, quantitative trait loci (QTL) were mapped using PI 90763 through two biparental F_3:4_ recombinant inbred line (RIL) populations segregating for *rhg1-a* and *rhg1-b* alleles against a SCN HG type 1.2.5.7 (Race 2) population. QTL located on chromosome 18 (*rhg1-a*) and chromosome 11 (*rhg2*) were determined to confer SCN resistance in PI 90763. The *rhg2* gene was fine-mapped to a 169-Kbp region pinpointing *GmSNAP11* as the strongest candidate gene. We demonstrated a unique epistatic interaction between *rhg1-a* and *rhg2* loci that not only confers resistance to multiple virulent SCN populations. Further, we showed that pyramiding *rhg2* with the conventional mode of resistance, *rhg1-b*, is ineffective against these virulent SCN populations. This highlights the importance of pyramiding *rhg1-a* and *rhg2* to maximize the impact of gene pyramiding strategies toward management of SCN populations virulent on *rhg1-b* sources of resistance. Our results lay the foundation for the next generation of soybean resistance breeding to combat the number one pathogen of soybean.

**Supplementary Information:**

The online version contains supplementary material available at 10.1007/s00122-022-04091-2.

## Introduction

Soybean cyst nematode (SCN; *Heterodera glycines*, Ichinohe) is a major threat to soybean production worldwide (Koenning and Wrather 2010; Allen et al. 2017; Tylka and Marett 2021). The monetary loss for soybean producers caused by SCN from 1996 through 2016 is estimated to be $32 billion with more than $1.5 billion in yield losses annually in the USA (Bandara et al. 2020). Current SCN management practices are based on an approach that includes planting-resistant cultivars, utilizing seed treatments, and implementing non-host crop rotations (Concibido et al. 2004; Niblack 2005; Mitchum 2016). Although plant genetic resistance is the most cost-effective and reliable management strategy, the genetic complexity of resistance poses a major barrier for breeding SCN resistance into new soybean cultivars (Niblack et al. 2008; Mitchum 2016). SCN management issues are further exacerbated by the existence of SCN populations with diverse virulence profiles (Niblack et al. 2002, 2008; Concibido et al. 2004; Tylka 2016). Therefore, a more complete understanding of known plant genetic resistance against modern virulent SCN populations is crucial for sustainable SCN management.

The pioneering discovery of *Rhg* genes (for Resistance to *H. glycines*) dates to the 1960s (Caldwell et al. 1960). Since then, there has been tremendous progress in understanding SCN genetic resistance through mapping of quantitative trait loci (QTL) from diverse soybean germplasm and the molecular characterization of some of these genes (Concibido et al. 2004; Mitchum 2016). Two major SCN resistance loci cqSCN-001 (*Rhg1*) and cqSCN-002 (*Rhg4*) were commonly mapped in different soybean germplasm and extensively utilized in the development of modern SCN-resistant soybean cultivars (Concibido et al. 2004; Liu et al. 2012, 2017; Cook et al. 2012, 2014; Mitchum 2016; Bayless et al. 2019). The classification of SCN resistance sources into plant introduction (PI) 88788 and Peking types was also primarily based on the presence of these two loci and their allelic variants (Brucker et al. 2005; Bayless et al. 2019). The *rhg1-b* resistance to SCN HG type 0 (Race 3) in PI 88788 is governed by a 31-kb repeated genomic region containing *GmSNAP18* (*α-soluble N-ethylmaleimide sensitive factor attachment protein*), one of the three genes contributing to SCN resistance (Cook et al. 2012, 2014; Lee et al. 2015). An epistatic interaction between the *rhg1-a SNAP18* allele and the *Rhg4* locus encoding a *serine hydroxymethyltransferase* (*SHMT08*) governs bi-genic resistance to SCN HG type 0 (Race 3) in Peking types such as the cultivar Forrest (Liu et al. 2012, 2017; Kandoth et al. 2017). However, these two resistance loci alone are incapable of explaining resistance mechanisms in all diverse soybean germplasm known to exhibit SCN resistance.

PI 88788 has been excessively utilized as a resistance source in modern SCN-resistant soybean cultivars due to resistance being derived from a single *rhg1-b* allele which allows for convenient breeding by introgression of a single resistance locus (Concibido et al. 2004; Niblack, 2005; Mitchum 2016; McCarville et al. 2017). Consequently, the monoculture of PI 88788-resistant soybean cultivars has facilitated the selection of virulent nematode populations that are capable of overcoming this resistance (Niblack et al. 2008; McCarville et al. 2017; Howland et al. 2018; Meinhardt et al. 2021). A crucial step to a successful SCN management strategy is the use of resistance sources capable of limiting the selection of virulent nematode populations which has often been undermined while breeding for high-yielding SCN-resistant cultivars (Niblack 2005; Chen 2020; Meinhardt et al. 2021). Due to the limited understanding of SCN virulence genes referred to as *ror* genes (for the reproduction on a resistant host), a strategic rotation of SCN resistance sources that can counter-select nematode populations is a potential solution for combating SCN (Gardner et al. 2017; Meinhardt et al. 2021). Counter-selection studies using different virulent nematode populations on PI 88788, PI 90763, and Peking have contributed to insights on the potential use of these sources in rotation to limit virulent SCN population build-up (Luedders and Dropkin 1983; Anand and Shumway 1985; Gardner et al. 2017; Meinhardt et al. 2021). Some of these studies have highlighted a strong counter-selection between virulence on PI 88788 and PI 90763 (Anand and Shumway 1985; Gardner et al. 2017). This has further elevated the unique resistance in PI 90763, which has been underutilized in traditional and modern cultivar development programs.

Several genetic mapping studies have reported approximately 216 SCN-resistant QTL from multiple sources on different chromosomes (SoyBase 2021). Confirmed SCN resistance QTL include *cqSCN-001*, *cqSCN-002*, *cqSCN-003*, *cqSCN-005*, *cqSCN-006*, and *cqSCN-007* (SoyBase 2021). The QTL *cqSCN-001* and *cqSCN-002* correspond to the *Rhg1* and *Rhg4* loci, respectively. Other SCN resistance QTL were mapped and confirmed, from multiple PIs and using multiple SCN populations, on Chr. 10 as *cqSCN10* (Vuong et al. 2010; Zhou et al. 2021), 11 as *cqSCN11/rhg2* (Wu et al. 2009; St. Amour et al. 2020; Suzuki et al. 2020), 15 as *cqSCN-006* (Kim and Diers 2013; Yu and Diers 2017), 16 as *cqSCN-003* (Concibido et al. 1997; Glover et al. 2004), 17 as *cqSCN-005* (Kazi et al. 2010), and 18 as *cqSCN-007* (Vuong et al. 2010; Kim and Diers 2013; Yu and Diers 2017; Usovsky et al. 2021a). PI 90763 is a highly resistant source against SCN populations with diverse virulent profiles (Anand and Shumway 1985; Arelli et al. 1997; Guo et al. 2005; Gardner et al. 2017). It has also been utilized as an indicator line in the SCN (HG) type test (Niblack et al. 2002). SCN resistance in PI 90763 to multiple SCN populations has been mapped to Chr. 18 by Concibido et al. (1997) using restriction fragment length polymorphism (RFLP) markers. Later, Guo et al. (2005) reported QTL on Chr. 6, 8, 11, 15, 16, 18, and 19 using *s*imple sequence repeat (SSR) markers and multiple SCN populations. However, limited research has been conducted to determine the impact of different known SCN resistance loci from PI 90763 and their interactions with *rhg1-b*. SCN resistance in PI 90763 is underutilized with limited resistance mapping studies and no reports on the incorporation of SCN resistance loci from PI 90763 into modern resistant cultivars to date (Kofsky et al. 2021). The fundamental reason behind the underutilization of the PI 90763 source is potentially attributed to its similar resistance response as Peking-type sources (Myers and Anand 1991; Concibido et al. 1997; Guo et al. 2005). Hence, the genetic resistance in PI 90763 can be a useful resource in unraveling different resistance mechanisms among Peking-type sources and decoding counter-selection of SCN virulence genes, which could aid in the design of a long-term SCN management strategy. Thus, the specific objectives of this study were to: (1) determine genomic regions governing SCN resistance in PI 90763 to SCN HG type 1.2.5.7 through linkage and nested association mapping (NAM) strategies, (2) fine map the *rhg2* gene to identify potential candidate genes, and (3) investigate the impact of resistance allele combinations against different SCN populations to devise an effective long-term SCN management strategy.

## Materials and methods

### Population development

To study SCN resistance in PI 90763 through genetic linkage analysis, we developed two F_3:4_ recombinant inbred line (RIL) mapping populations by crossing resistant parents with contrasting *rhg1-a* and *rhg1-b* alleles at the *Rhg1* locus. The usefulness of F_3_-derived populations in genetic mapping studies has been previously established (Takuno et al. 2012). The first mapping population (pop1) was developed by crossing high-yielding elite soybean line SA13-1385 (*rhg1-b;* PI 88788 type) with PI 90763 (*rhg1-a*). Similarly, the second mapping population (pop2) was created by crossing the high-yielding elite soybean line LD11-2170 (*rhg1-b;* PI 88788 type) and PI 90763 (*rhg1-a*). A third population developed by a cross between a susceptible soybean line SA10-8471 (pop3) and PI 90763 (*rhg1-a*) was used along with pop1 and pop2 for the NAM analysis.

PI 90763 is highly resistant to multiple SCN populations (Arelli et al. 1997; Klepadlo et al. 2018), whereas SA13-1385 and LD11-2170 carry PI 88788-type resistance and were released as SCN-resistant varieties from the University of Missouri-Columbia and the University of Illinois, Urbana-Champaign, respectively. SA13-1385 and LD11-2170 are highly resistant to HG type 0. A total of 330, 274, and 218 RILs were developed for pop1, pop2, and pop3, respectively. The cross-pollination for pop1 and pop2 were made at the Bay Farm Research Facility in Columbia, MO during the summer of 2017. The RIL populations were inbred and advanced using the single-seed descent method (Brim 1966) at Hartung Brothers Inc. winter nursery in Kekaha, HI. Hybrid F_1_ seeds were harvested during the summer of 2017 and were sent to Kauai, Hawaii, for advancing generations at the winter nursery in October 2017. The cross-pollination for pop 3 was made at the Bay Farm Research Facility in Columbia, MO, during the summer of 2019, and F_3:4_ RILs from pop3 were similarly developed.

### SCN bioassay

The soybean cyst nematode bioassay was conducted following the standardized cyst evaluation protocol (Niblack et al. 2009). The SCN inbred population TN22, HG type 1.2.5.7 (Race 2) was used as the inoculum source. Five seedlings from each F_3:4_ line were transplanted along with parental lines, the susceptible checks Lee 74 and Williams 82, Pickett, and the HG type test indicator lines (PI 548402, PI 88788, PI 90763, PI 437654, PI 209332, PI 89772, and PI 548316) (Niblack et al. 2002). Each mapping population was tested at a different period due to limited greenhouse capacity. The seedlings were inoculated with 1200 eggs two days after transplanting. Each of five replicated seedlings per experimental line was planted in a different micro-pot and organized in a randomized complete block design. A root temperature of 27 °C was maintained throughout the experiment. Thirty days after inoculation, roots were soaked in water to remove the soil and sprayed with high-pressure water over a set of nested sieves (no. 20 over no. 60). The cysts obtained from each plant root system were manually counted under a stereoscope, and an average number of cysts for each line was determined. Female indices (FI) were calculated for each line by dividing the average number of cysts on each line by the average number of cysts on the susceptible parent and multiplying by 100 (Nibalck et al. 2002). Experimental lines were rated following a standardized method as resistant (R, FI < 10%), moderately resistant (MR, FI = 10–30%), moderately susceptible (MS, FI = 31–60%), and susceptible (S, FI > 60%) (Schmitt and Shannon 1992). Shapiro–Wilk's test was performed in RStudio to determine the normality of distribution of female indices while symmetry was analyzed using skewness and kurtosis of the distributions.

### Genotyping and SNP analysis

Genomic DNA was extracted with the cetyl trimethyl ammonium bromide (CTAB) method (Doyle and Doyle 1987). 10 young trifoliate leaves from 10 to 15 plants of each F_3:4_ line were bulked, freeze-dried and DNA samples were extracted. The extracted DNA samples from the three populations were submitted to the Soybean Genomics and Improvement Laboratory, USDA-ARS, Beltsville, MD, for single-nucleotide polymorphism (SNP) genotyping using the Illumina Infinium BARCSoySNP6K BeadChip (Song et al. 2020), and allele calls were made using Genome Studio software (Illumina Inc). SNP markers obtained from genotyping using the Illumina Infinium BARCSoySNP6K BeadChip for both populations were filtered using TASSEL software (Bradbury et al. 2007). The RILs exceeding more than 10% of missing calls and 30% heterozygosity were eliminated. The SNP matrix was then converted to A/B/H format for both the populations individually using ABH genotype in TASSEL (Bradbury et al. 2007). The ABHgenotypeR package was used in RStudio to conduct an imputation of the missing genotypes based on flanking alleles (Reuscher and Furuta 2016). A similarity test was conducted to eliminate RILs below 90% genetic similarity between the parents using the R package “ParentOffSpring” (Abdel-Haleem et al. 2013). Obtained SNP matrixes were utilized for constructing genetic linkage maps using the qtl package in RStudio (Browman and Sen 2009).

### Genetic linkage and nested association mapping

Mapping of quantitative trait loci was performed using MapQTL 5.0 (van Ooijen 2004). Interval mapping (IM) at 1-cM intervals along the chromosome was used to detect QTL based on a LOD threshold of 3.0. This corresponds to a false discovery rate (FDR) of 0.05, which was determined by permutation tests. Markers closely linked to positions with the highest LOD scores were taken as cofactors for multiple-QTL modeling (MQM) analysis (van Ooijen 2004). Graphical presentation of QTL was drawn using MapChart 2.3 (Voorrips 2002). The composite interval mapping (CIM) through the RStudio qtl package was performed for the further confirmation of QTL mapping results obtained from the MapQTL (Browman and Sen 2009).

Nested Association Mapping was conducted through the NAM package in RStudio with an efficient mixed-model association algorithm (Xavier et al. 2015) using the three F_3:4_ RIL populations for NAM analysis. To determine the significance of SNPs in the association mapping, an FDR threshold at α ≤ 0.001 was calculated, and SNPs were declared significant based on the FDR threshold value.

### Fine-mapping of *rhg2* locus

Twenty-four F_3:5_ lines homozygous at *rhg1-a* and heterozygous at the *rhg2* from pop1 were selected based on confidence intervals delimited in this study and confirmed using two KASP assays (Kompetitive Allele Specific PCR): Rhg1-2 and SNAP11 (Kadam et al. 2016; Usovsky et al. 2021a). The presence/absence of the *Rhg4* resistance allele was confirmed by KASP assay using Rhg4-5 marker (Kadam et al. 2016). Twenty plants from each of the selected lines were tagged individually, young trifoliate leaves were collected and freeze-dried, and DNA was extracted following the modified CTAB method as previously described. Four KASP assays (Rhg1-2, SNAP18-1, SNAP11-1, and Rhg4-5) were used to confirm the homozygosity state of each F_5:6_ plant. A set of 24 KASP assays (named as “MU”) were newly designed based on the whole-genome sequencing data to detect the recombination spots near the *rhg2* locus (www.SoyBase.org). The F_5:6_ lines were phenotyped with SCN population TN22 (HG type 1.2.5.7) with five replications following standardized procedures (Niblack et al. 2009).

### SCN screening of allelic combinations

Ninety-two F_3:5_ lines from pop1 homozygous at *Rhg1*, *rhg2*, and *Rhg4* were selected based on confidence intervals delimited in this study, which was confirmed using four Kompetitive Allele Specific PCR (KASP) assays: Rhg1-2 (For *rhg1*), SNAP18-1 (For *GmSNAP18*), SNAP11-1 (For *GmSNAP11*, and Rhg4-5 (For *Rhg4*) (Kadam et al. 2016; Usovsky et al. 2021a). These lines were separated into eight different categories based on their allelic combinations: A1 = *rhg1-a* (*n* = 15)*,* A2 = *rhg1-a* + *rhg2* (*n* = 10), A3 = *rhg1-a* + *rhg2* + *Rhg4* (*n* = 5), A4 = *rhg1-a* + *Rhg4* (*n* = 21), B1 = *rhg1-b* (*n* = 4)*,* B2 = *rhg1-b* + *rhg2* (*n* = 17), B3 = *rhg1-b* + *rhg2* + *Rhg4* (*n* = 15), and B4 = *rhg1-b* + *Rhg4* (*n* = 5). Similarly, 69 F_3:5_ lines were selected from pop2 where only four of the above allelic combinations were present as *rhg2* was not segregating. These four allelic combinations included: A2 = *rhg1-a* + *rhg2* (*n* = 14), A3 = *rhg1-a* + *rhg2* + *Rhg4* (*n* = 16)*,* B2 = *rhg1-b* + *rhg2* (*n* = 15), and B3 = *rhg1-b* + *rhg2* + *Rhg4* (*n* = 24).

Selected RILs with different allelic combinations for pop1 and pop2 were screened against SCN inbred populations TN7 (HG type 2.5.7; race 1), TN22 (HG type 1.2.5.7; race 2), PA3 (HG type 0; race 3), and MM4 (HG type 2.5.7; race 5). Screening of selected RILs in pop1 and pop2 was conducted at two different periods with five replications in a completely randomized design. SCN inoculations and data collection were conducted based on the standardized screening procedure as previously described. Female indices calculated from the screening of the allelic combinations from both populations were analyzed by one-way analysis of variance (ANOVA) in RStudio, and Tukey’s HSD test was used for multiple comparisons of SCN phenotypes with different allelic combinations.

## Results

### Phenotypic variation of SCN resistance and genetic linkage mapping

**Fig. 1:**
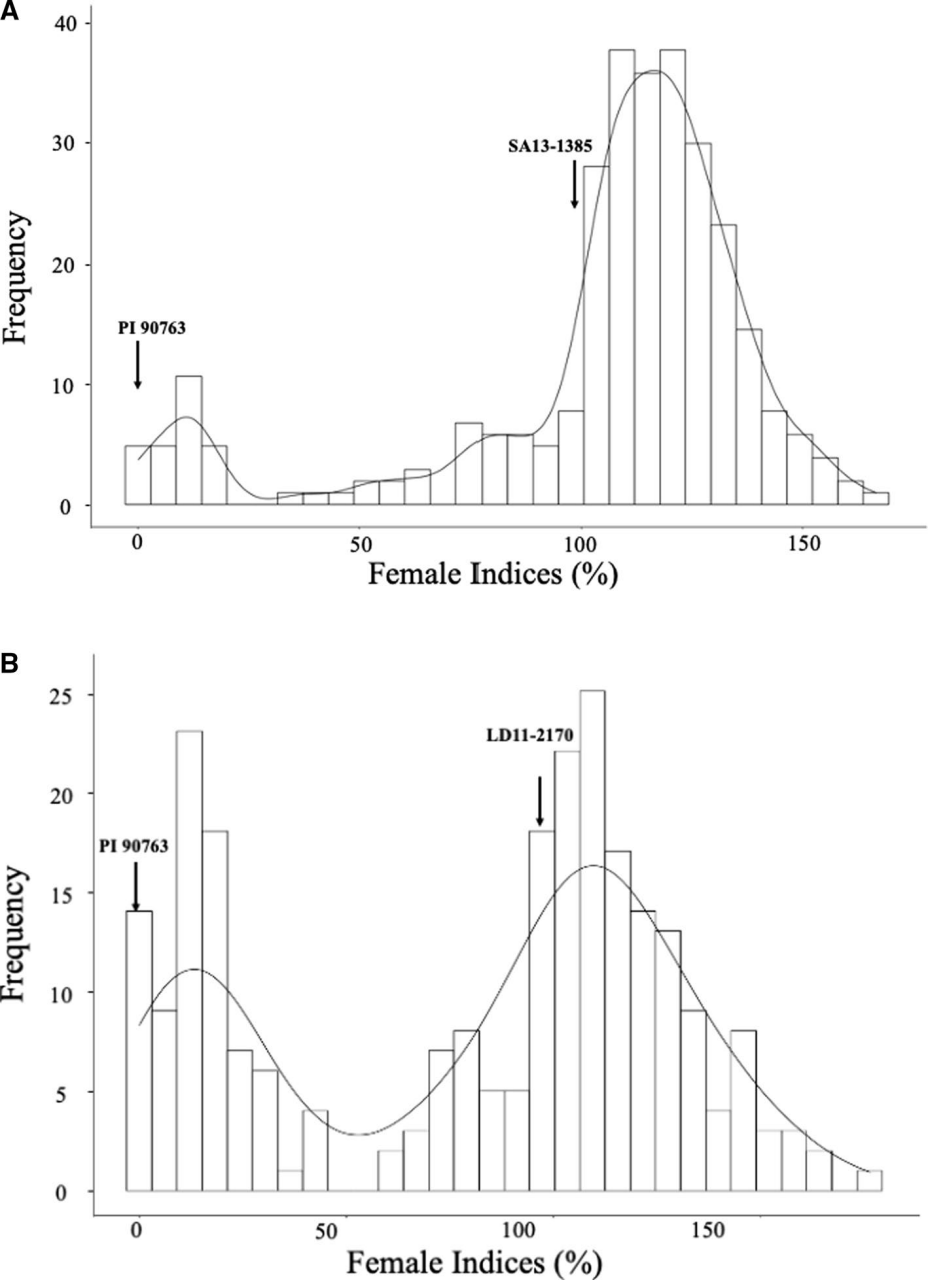
Frequency distribution of female indices against SCN population TN22 (HG type 1.2.5.7). **A** 303 F_3:4_ RILs from pop1 (SA13-1385 × PI 90763) and **B** 251 F_3:4_ RILs from pop2 (LD11-2170 × PI 90763)

Two biparental mapping populations were screened against the TN22 SCN population to map resistance QTL regions. Additionally, seven HG type indicator lines, two susceptible lines, and one race differential line confirmed the correct responses of HG type 1.2.5.7 (race 2) (Supplemental Table 1). The FI of parental lines for pop1 was 83 and 0 for SA13-1385 and PI 90763, respectively (Fig. 1A; Table 1). For pop2, the FI of parental lines LD11-2170 and PI 90763 were 94 and 0, respectively (Fig. 1B; Table 1). Therefore, both of the parents SA13-1385 and LD11-2170 were highly susceptible and PI 90763 was highly resistant against SCN population TN22. The phenotypic distribution of FI in pop1 ranged from 0 to 167 with a mean of 106 based on the susceptible parent SA13-1385 (Fig. 1a; Table 1), whereas the phenotypic distribution of FI in pop2 ranged from 0 to 176 with a mean of 80 based on the susceptible parent LD11-2170 (Fig. 1b; Table 1). The differences in frequency distributions indicated divergent genetic backgrounds for SCN resistance in pop1 and pop2.

**Table 1:** Descriptive statistics of female indices of 303 F_3:4_ lines from pop1 (SA13-1385 × PI 90763) and 251 F_3:4_ lines from pop2 (LD11-2170 × PI 90763) as their responses to SCN population TN22 (HG type 1.2.5.7)

Mean cysts			Female Index [%]				Shapiro–Wilk (*w*)	Skewness	Kurtosis
			303 F_3:4_ (pop1)						
Williams 82	SA13-1385	PI 90763	Mean	Min	Max	SD			
209	173	0	106	0	167	49.1	0.81	−1.63	5.3

			251 F_3:4_ (pop2)						
Williams 82	LD11-2170	PI 90763	Mean	Min	Max	SD			
252	236	0.4	80	0	176	35.1	0.89	−0.38	1.7

The parental lines were added to evaluate resistance differences. The normality tests of the female index (%) are shown by Shapiro–Wilk (*w*), skewness, and kurtosis

**Fig. 2:**
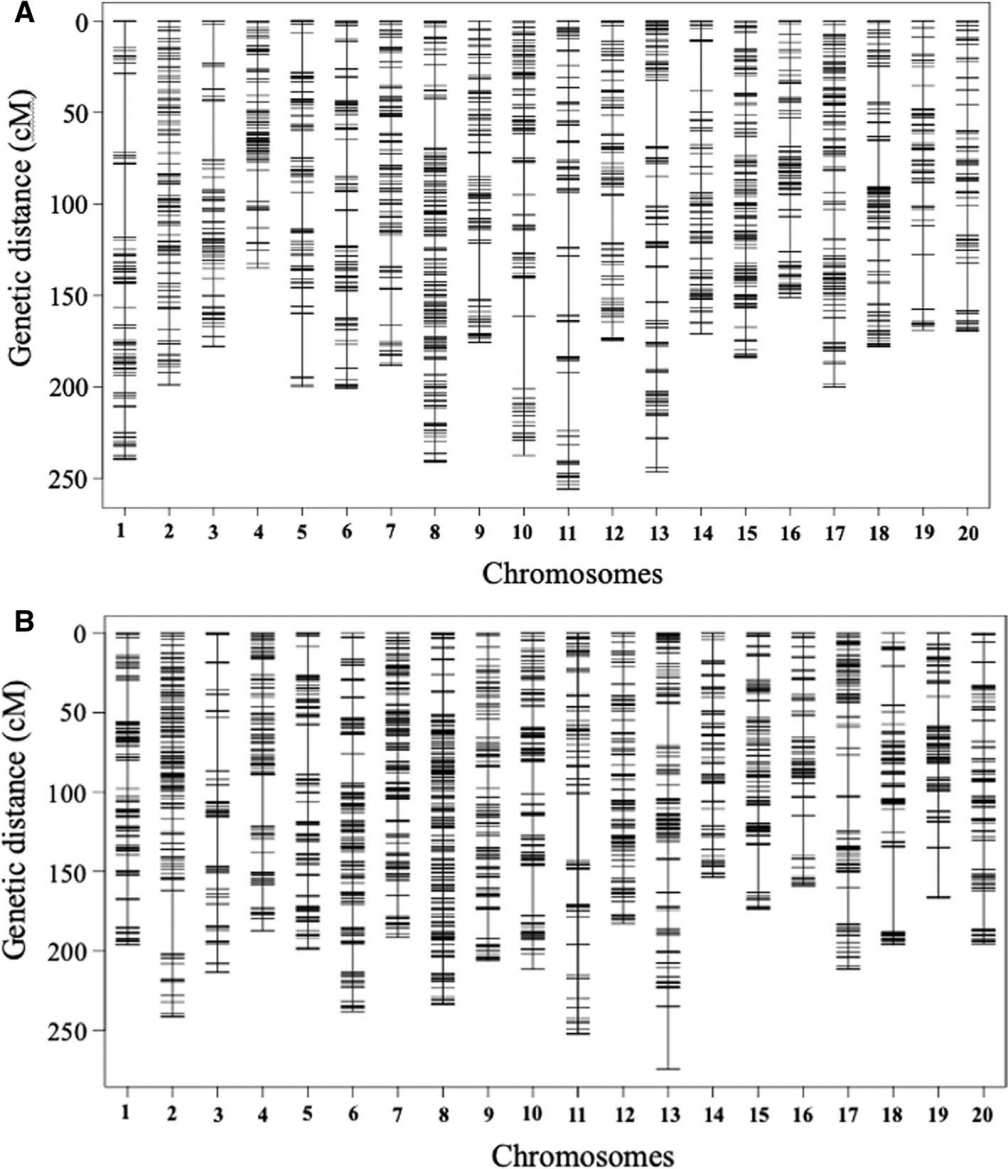
Genetic linkage map created for **A** 303 F_3:4_ RILs from pop1 (SA13-1385 and PI 90763) and **B** 251 F_3:4_ RILs from pop2 (LD11-2170 × PI 90763). The *X*-axis represents chromosome numbers, and *Y*-axis represents the genetic position of single-nucleotide polymorphism (SNP) markers. Distribution and SNPs are represented by black bars across each chromosome

**Fig. 3:**
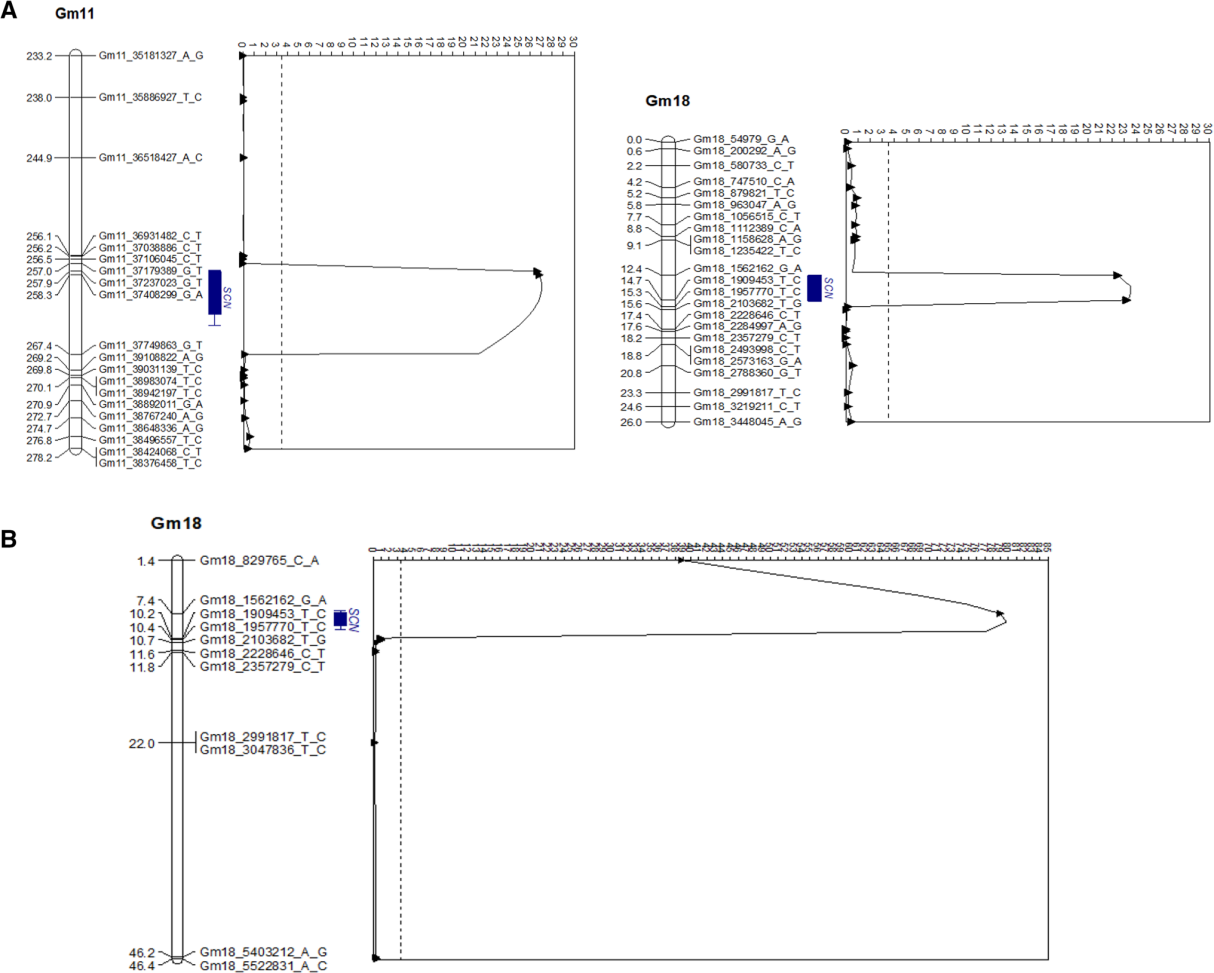
Quantitative trait loci (QTL) controlling soybean cyst nematode (SCN) resistance to TN22 (HG type 1.2.5.7) in PI 90763: **A** two major QTL detected in pop1 (SA13-1385 × PI 90763); **B** one major QTL detected in pop2 (LD11-2170 × PI 90763). Scales on the left of the chromosome represent the map position in centiMorgans (cM). Scales on the top of the graph represent the value of the logarithm of the odds (LOD). The black dotted line indicates the threshold of significance (LOD = 3.5 and 3.4) for pop1 and pop2, respectively

Genetic linkage maps for both populations were constructed using the qtl package in RStudio based on the segregation of SNP markers across 20 chromosomes. Approximately 6000 raw SNPs were obtained from the Illumina Infinium SoySNP6K assay for each population. The number of high-quality polymorphic markers showing distinct segregation that were obtained after filtering was 2265 for pop1 and 2123 for pop2 (Fig. 2A, B; Table 2). SNP markers spanned through 4177.7 and 4090.3 cM for pop1 and pop2, respectively, and both linkage maps provided comparable coverage of SNPs in both populations (Table 2; Fig. 2). Two major QTL on Chr. 11 and 18 were detected by the multiple-QTL modeling (MQM) method in pop1 (Fig. 3A; Table 3). The QTL on Chr. 11 and 18 explained 28.3 and 23.9% of total phenotypic variance and their additive effects were 23.2 and 21.5, respectively, and PI 90763 was the source of resistance alleles for both. The Chr. 11 QTL was mapped to a 575-kbp interval between the markers Gm11_37237023—Gm11_37749863 (Wm82.a2. v1) with a peak at Gm11_37408299 marker (Gm11:32959788; Wm82.a2. v1). The Chr. 18 QTL was mapped to a 374-kbp interval between the markers Gm18_1562162 and Gm18_1909453 (Wm82.a2. v1) with a peak at Gm18_1909453 marker (Gm18:1909982; Wm82.a2. v1) (Fig. 3, Table 3). CIM analysis performed in RStudio using the qtl package detected both QTL along with their significant epistatic interaction (Supplemental Fig. 1A; Supplemental Table 2; 3A).

**Table 2:** Summary of 20 genetic linkage groups developed from (A) 303 F_3:4_ lines of pop1 (SA13-1385 × PI 90763), and (B) 251 F_3:4_ lines of pop2 (LD11-2170 × PI 90763)

Chr	No. SNPs	Length	Average Interval	Max Space	No. SNPs	Length	Average Interval	Max Space
	pop1	cM	cM	cM	pop2	cM	cM	cM
1	103	241.5	2.4	37	99	202.7	2.1	30.2
2	126	211.5	1.7	15.4	133	240.6	1.8	40.5
3	82	192.1	2.4	35.8	67	212.0	3.2	31.1
4	94	156.5	1.7	20.3	102	197	2.0	32.4
5	100	219.2	2.2	32	102	198.9	2.0	31.3
6	111	221.4	2.0	20.4	133	236.1	1.8	18.4
7	119	197.1	1.7	21.3	141	190.1	1.4	13.7
8	205	253.8	1.2	22.6	185	230.9	1.3	14.4
9	100	194.1	2.0	27.6	112	207.7	1.9	18.9
10	129	236.1	1.8	31.9	117	211.1	1.8	32.3
11	103	277.6	2.7	43.4	88	250.9	1.9	42.1
12	99	196.8	2.0	26.3	99	181.4	2.3	11.0
13	127	268.5	2.1	42.8	122	276.6	2.0	39.0
14	92	179.9	2.0	28.9	76	153.2	2.3	12.3
15	149	200.5	1.4	13.8	115	173.6	1.5	26.6
16	103	162.3	1.6	24.2	69	158.3	2.3	26.0
17	133	209.8	1.6	16.4	112	209.8	1.9	25.9
18	121	190.6	1.6	24.3	79	194.5	2.5	51.8
19	81	185.2	2.3	34.9	82	165.2	2.0	30.1
20	88	183.4	2.1	24.6	90	200.2	2.2	24.4
Total	2265	4177.7	1.9	43.4	2123	4090.3	1.9	41.8

**Table 3:** Quantitative trait loci (QTL) for resistance to SCN population TN22 (HG type 1.2.5.7) mapped in 303 F_3:4_ lines of pop1 (SA13-1385 × PI 90763) and 251 F_3:4_ lines of pop2 (LD11-2170 × PI 90763)

Population	Peak Marker	Peak Position^a^	CI Markers	CI Position^a^	CI Size	LOD	PV^b^	Add
Pop1	Gm11_37408299	32959788	Gm11_37237023–Gm11_37749863	3784991–3330696	575 Kbp	27.1	28.3%	23.2
	Gm18_1909453	1909982	Gm18_1562162–Gm18_1909453	1562536–1909982	347 Kbp	23.5	23.9%	21.5
Pop2	Gm18_1562162	1562536	Gm18_829765–Gm18_1909453	830106–1909982	1080 Kbp	79.8	76.4%	50.1

CI Confidence interval; *LOD* Logarithm of odds; *PV* Percentage of variation, *Add* Additive effects

^a^Peak and confidence interval physical position based on Wm82.a2. v1

^b^Percentage of phenotypic Variation represented by QTL

A single major QTL was mapped to Chr. 18 using the MQM method in pop2 which explained 76.4% of the total phenotypic variation (Fig. 3B; Table 3). The additive effect for the QTL was 50.1 with a resistance allele originating from PI 90763 (Table 3). The QTL spanned through the 1080-Kbp region where the confidence interval was established between the markers Gm18_829765 and Gm18_1909453 that corresponded to a physical location of Gm18:830106–1909982 (Wm82.a2.v1) with a peak at Gm18_1562162 marker (Gm18:1562536; Wm82.a2.v1) (Fig. 3B; Table 3). CIM analysis performed using qtl package in RStudio detected the same QTL (Supplemental Fig. 1B; Supplemental Table 2; 3B). The genomic regions of Chr. 18 in both the mapping populations correspond to the *Rhg1* locus, whereas the *rhg2* gene nomenclature is now associated with the genomic region of the QTL on Chr. 11. Although *rhg2* has been detected in a few other resistance sources, the underlying gene(s) have not been cloned. Hence, the genetic linkage analysis revealed *rhg1-a* and *rhg2* resistance loci govern resistance against the SCN inbred population TN22 (HG type 1.2.5.7).

### Nested association mapping

**Fig. 4:**
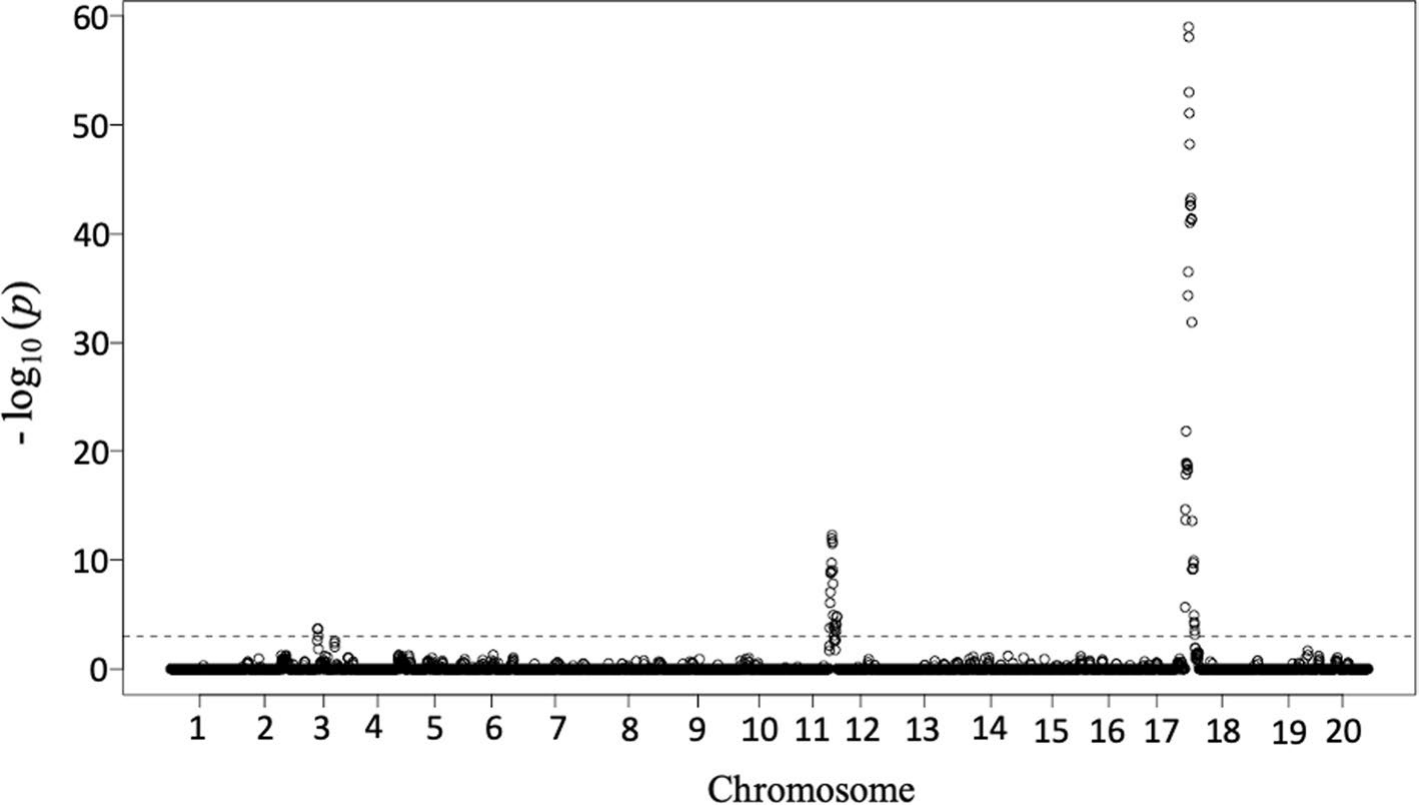
Manhattan plots of nested association mapping (NAM) analysis for SCN population TN22 (HG type 1.2.5.7) plotted against positions on each of the 20 chromosomes. The NAM panel included pop1 (SA13-1385 × PI 90763), pop2 (LD11-2170 × PI 90763) and pop3 (SA10-8471 × PI 90763). The significant SNPs were distinguished by the false discovery rate (FDR) of the α ≤ 0.001 thresholds represented by the dotted line

SNP markers with the greatest LOD score for resistance to the TN22 population (HG type 1.2.5.7) were identified in the NAM analysis. Additive allelic effects were determined relative to the hub parent PI 90763 where a positive effect represented an increase in FI due to allele substitution of PI 90763 alleles by alleles from the founder parents. A negative effect represented a reduction in FI due to allele substitution of founder parent allele with an allele from PI 90763. Using a false discovery rate threshold of α ≤ 0.001, several SNPs associated with SCN resistance were identified from the NAM analysis (Table 4) and these mapped to the Chr. 11 and 18 intervals identified by the MQM analysis. However, the significance of QTL detected through the NAM population in Chr. 11 was lower than that in pop1 using the MQM analysis (Fig. 4; Supplementary Fig. 1A). This depicts that the allele frequency in mapping populations primarily contributes to the significance of QTL in NAM analysis (Yu et al. 2008; Beche et al. 2020). The NAM analysis further detected an additional QTL on Chr. 03 using three mapping populations (Fig. 4; Table 4), whereas only two QTL peaks were detected in pop3 in Chr.11 and 18 (data not shown) through the linkage analysis. These data further corroborate the power of rare allele detection in the NAM analysis as observed with increased detection of trait-associated markers (Yu et al. 2008; Beche et al. 2020). The top three significant SNPs for each QTL obtained from the NAM analysis are listed in Table 4. SCN resistance QTL on Chr. 03 was previously determined for HG type 2.5.7 (race 1) in PI 90763 and HG type 0 (race 6) in PI 209332 (Concibido et al. 1997), HG type 2.5.7 (race 5) on PI 404198A (Guo et al. 2006), HG type 1.3.5.6.7 and HG type 1.2.5.7 in PI 437655 (Jiao et al. 2015). Thus, the NAM analysis reaffirmed the QTL obtained from the linkage mapping analysis along with an additional QTL on Chr. 03 against SCN population TN22 (HG type 1.2.5.7).

**Table 4:** Summary of significant SNPs from NAM analysis for SCN resistance against TN 22 (HG type 1.2.5.7) and their respective allelic effect with negative and positive effects relative to the common parent (PI 90763)

SNP ID	Chr	Allele	−log (10)*P*	Position	Allelic effects			
					PI 90763	SA13-1385	LD11-2170	SA10-8471
ss715629539	18	T/C	58.97	1909453	−17.23	−5.20	26.60	−4.17
ss715629620	18	T/C	58.05	1957770	−16.68	−5.54	26.80	−4.58
ss715629684	18	G/T	52.99	2007638	−16.39	−5.44	26.61	−4.78
ss715610409	11	G/T	12.28	37338181	−6.87	9.04	−8.28	6.11
ss715610387	11	G/T	11.95	37237023	−6.77	8.76	−8.19	6.20
ss715610424	11	C/T	11.70	37462158	−7.47	9.46	−8.04	6.04
ss715585098	3	T/C	3.69	30809885	−3.07	6.47	−1.62	−1.78
ss715585092	3	T/C	3.66	30647053	−3.25	6.08	−0.97	−1.87
ss715585107	3	A/G	3.65	30955790	−3.05	6.43	−1.61	−1.77

### Fine-mapping of *rhg2* gene

**Fig. 5:**
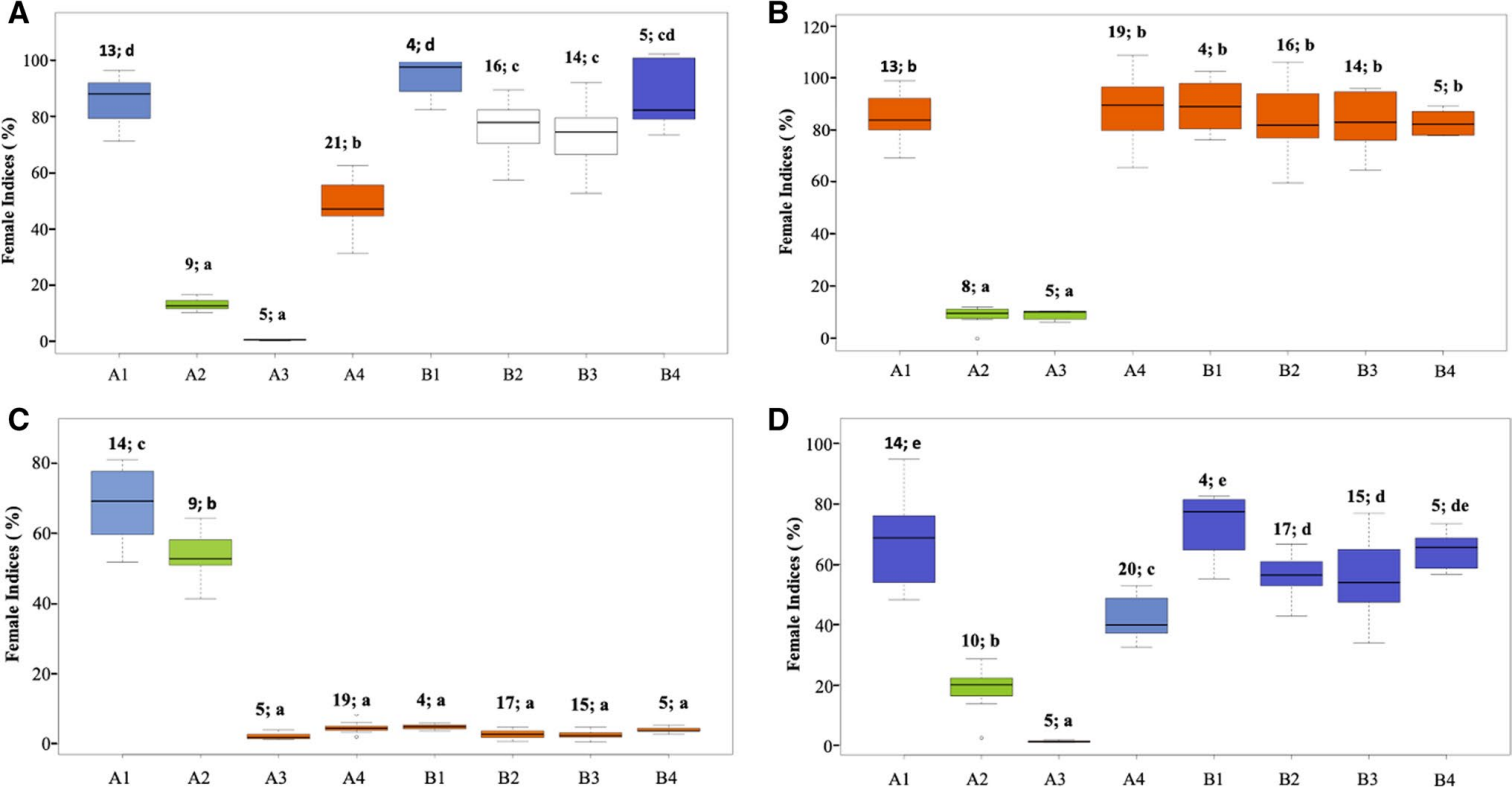
Phenotypic responses of lines carrying eight allelic combinations from pop1 (SA13-1385 × PI 90763) tested against SCN populations **a** TN7 (HG type 2.5.7; race 1), **b** TN22 (HG type 1.2.5.7; race 2), **c** PA3 (HG type 0; race 3), and **d** MM4 (HG type 2.5.7; race 5). The *X*-axis represents different allelic combinations of homozygous SCN resistance alleles/QTL. The numbers and letters above each box plot indicate the number of lines tested and significance grouping based on Tukey's honest significant difference test (Tukey's HSD) at *P* ≤ 0.05. Legend: **A1** = *rhg1-a*; **A2** = *rhg1-a* + *rhg2*; **A3** = *rhg1-a* + *rhg2* + *Rhg4*; **A4** = *rhg1-a* + *Rhg4*, **B1** = *rhg1-b*; **B2** = *rhg1-b* + *rhg2*; **B3** = *rhg1-b* + *rhg2* + *Rhg4*; **B4** = *rhg1-b* + *Rhg4*

Fine-mapping of the *rhg2* gene was conducted to narrow the genetic region and pinpoint candidate genes imparting SCN resistance. Twenty F_3:5_ RILs from pop1 were pooled from RILs with both the loci where *rhg1-a* was homozygous and *rhg2* was homozygous at one end and heterozygous at the other end of the confidence interval determined by QTL mapping (Table 3). These lines were advanced to create F_5:6_ sister lines for each recombination event within the *rhg2* region. Six crossing-over events were identified in lines SA18-17394, SA18-17486, SA18-17447, SA18-17176, SA18-17229, and SA18-17370 (Fig. 5). The fine-mapping analysis further delimited the *rhg2* gene between MU-35 and MU-52 markers indicating that *rhg2* is in the 169-Kbp interval between MU-35 and MU-52 (Gm11:32906157–33075108; Wm82. a.2.v.1) (Supplemental Table 4). There are 21 potential candidate genes within the fine-mapped region based on the Williams 82 reference genome (www.SoyBase.org). The strongest candidate was found to be *GmSNAP11* gene, a paralog of the *GmSNAP18* gene at the *Rhg1* locus (Cook et al. 2012, p. 14; Lakhssassi et al. 2017; St-Amour et al. 2020; Usovsky et al. 2021b). This result further suggests that PI 88788-type *GmSNAP18*, Peking-type *GmSNAP18*, and their paralog *GmSNAP11* have evolved to underlie different types of resistance and/or diversified function of a pleiotropic role of Peking-type *GmSNAP18* and *GmSNAP11* as in reniform nematode resistance (Usovsky et al. 2021b). Our mapping of *the rhg2* locus to a 169-Kbp interval is smaller than the 821-Kbp region; it was previously mapped to (Suzuki et al. 2020). Hence, the *rhg2* gene was fine-mapped to 169-Kbp region and the *GmSNAP18* gene was identified as the potential candidate gene.

### SCN screening of specific allelic combinations of *rhg1-a, rhg1-b, rhg2*, and *Rhg4* loci

**Fig. 6:**
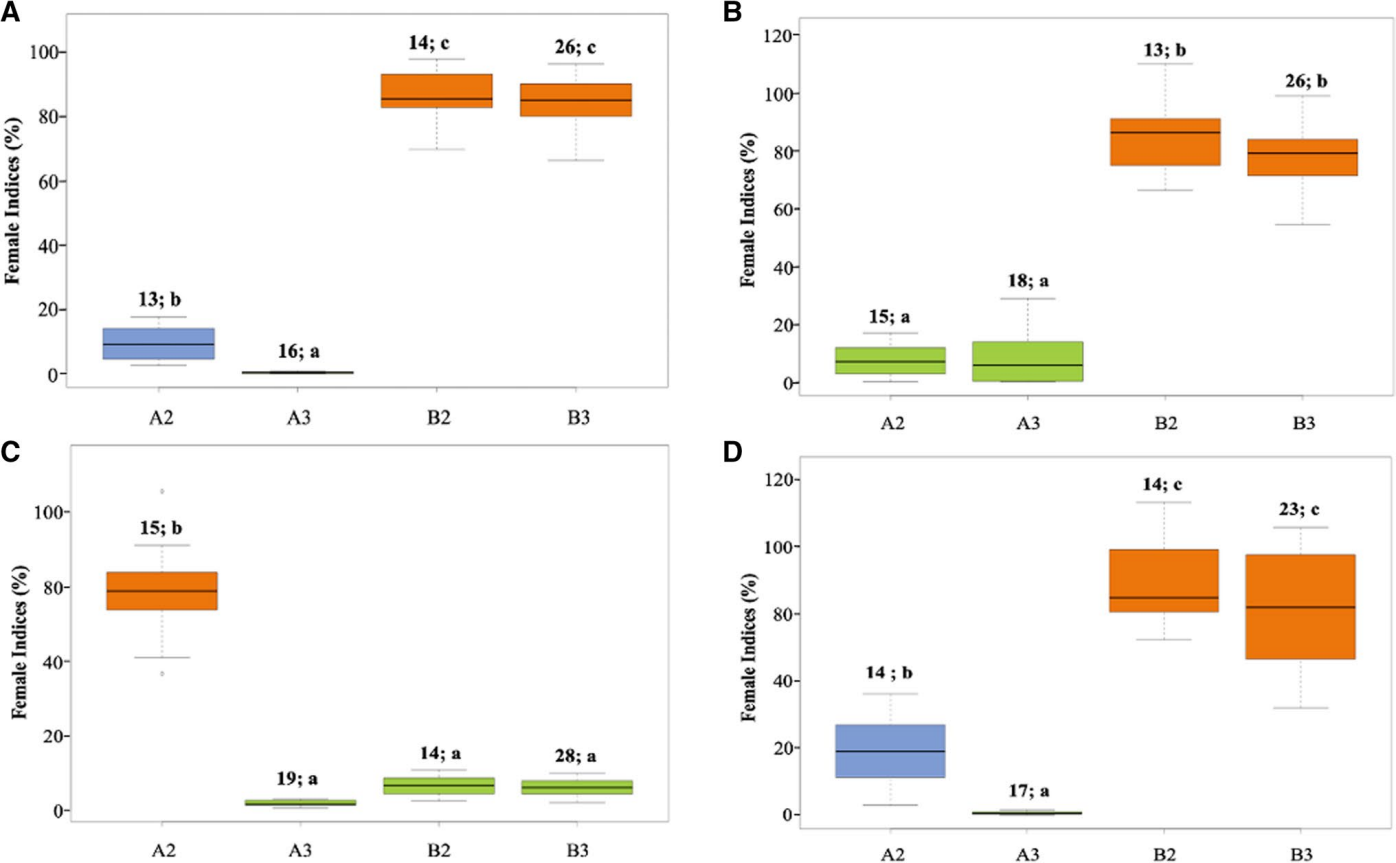
Phenotypic responses of lines carrying four allelic combinations from pop2 (LD11-2170 × PI 90763) tested against SCN populations **A** TN7(HG type 2.5.7; race 1), **B** TN22 (HG type 1.2.5.7; race 2), **C** PA3 (HG type 0; race 3), and **D** MM4 (HG type 2.5.7; race 5). The *X*-axis represents different allelic combinations of homozygous SCN resistance alleles/QTL. The numbers and letters above each box plot indicate the number of lines tested and significance grouping based on Tukey's test (Tukey's HSD) at *P* ≤ 0.05

**Fig. 7:**
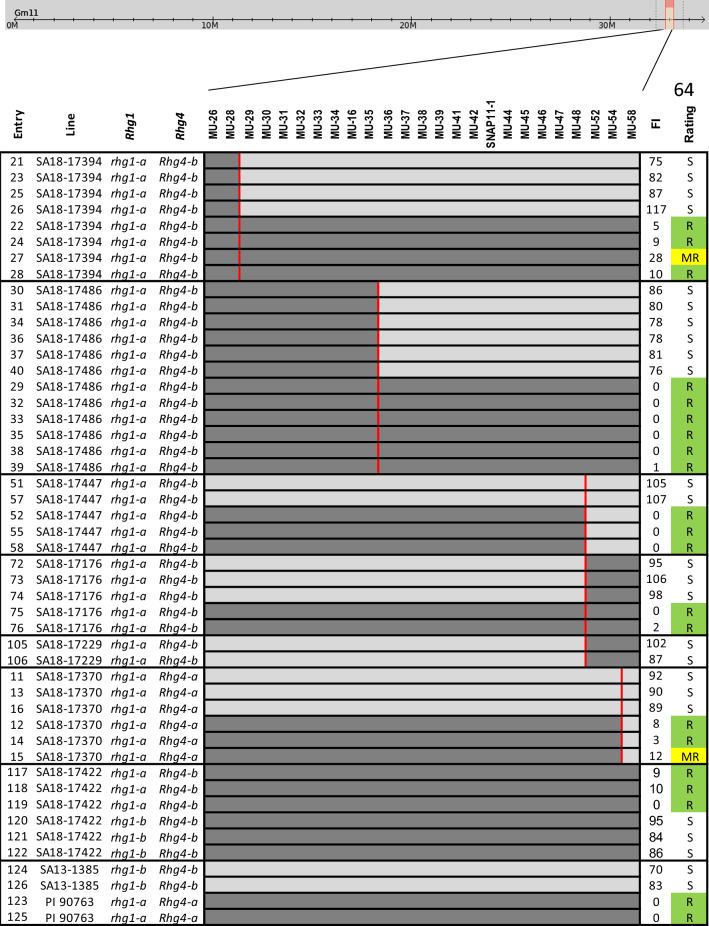
Fine-mapping of *rhg2* gen*e* in PI 90763. Alleles of *Rhg1* locus (resistant Peking-type *rhg1-a* vs. PI 88788-type *rhg1-b*) and *Rhg4* locus (resistant Peking-type *Rhg4-a* vs. susceptible Williams 82-type *Rhg4-b*) were confirmed using KASP assays (Kadam et al. 2016). Dark and light gray colors correspond to genomic regions derived from PI 90763 and SA13-1385, respectively. Vertical red lines signify recombination events between sister lines. Resistance rating to TN22 population (HG type 1.2.5.7) was calculated based on female index (FI): R = resistance (FI < 10), MR = moderate resistance (FI, 10–30), S = susceptibility (FI > 60)

SCN screening of different *Rhg* combinations demonstrated their impact against different SCN populations. Subgroups with *rhg1-a* + *rhg2* + *Rhg4* (FI = 0.6; Tukey’s HSD mean separation (MS) = *a*) and *rhg1-a* + *rhg2* (FI = 13, MS = *a*) in pop1 had similar FI and were the most resistant among all the combinations for TN7 (HG type 2.5.7) (Fig. 6A). Similar SCN phenotypic responses were observed for TN22 (HG type 1.2.5.7) and MM4 (HG type 2.5.7) SCN populations for *rhg1-a* + *rhg2* + *Rhg4* (FI = 11, MS = *a*; FI = 1, MS *a*) and *rhg1-a* + *rhg2* (FI = 8, MS = *a*; FI = 19, MS = *b*), respectively (Fig. 6B, D). The SCN phenotypic responses were validated by screening resistance loci combinations from pop2 which resulted in similar responses observed previously for pop1 (Fig. 7). The results confirmed the epistatic interaction of the *rhg1-a* and *rhg2* loci contributing resistance against TN7, TN22, and MM4. Consistent with prior studies, either *rhg1-b* alone or a combination of *rhg1-a* and *Rhg4* was sufficient for resistance to SCN HG type 0 (Race 3) (Figs. 6C, 7). The other combinations including *the rhg1-b* allele displayed moderately susceptible or susceptible phenotypic responses against the three SCN populations (Fig. 6a, b, d). Thus, the results highlight that a pyramid of the *rhg1-a* allele with *rhg2* offers an advantage against the virulent SCN populations tested. Since *rhg1-b* is the most widely used resistance allele in more than 95% of resistant soybean cultivars planted in the USA, current efforts have focused on pyramiding of *rhg1-b* with other resistance loci to enhance resistance to virulent SCN (Brzostowski and Diers 2017; Yu and Diers 2017; Meinhardt et al. 2021). Here, we demonstrated that a combination of *rhg1-a* and *rhg2* genes provides resistance against virulent nematode populations and resistance allele pyramid focused on these genes would be an effective strategy for the management of virulent SCN populations.

## Discussion

In this study, we demonstrated an epistatic interaction between *rhg1-a* and *rhg2* loci in PI 90763, imparting SCN resistance against virulent SCN populations using two unique mapping populations created by a cross between *rhg1-b* (SA13-1385/LD11-2170) and *rhg1-a* (PI 90763). We mapped *rhg1-a* and *rhg2* in pop1, whereas only *rhg1-a* was mapped in pop2. Further KASP assays on parents determined that the *rhg2* gene was present in the parent LD11-2170, which explained the detection of a single QTL on Chr. 18 in pop2. NAM analysis further confirmed the status of *rhg2* allele frequency in NAM mapping populations along with an additional QTL at Chr. 03. The significant *rhg2* gene detected through genetic linkage and NAM analysis was further fine-mapped to a 169-kbp region using a genetic mapping approach. *GmSNAP11* was determined to be the strongest candidate gene. Previous reports on cloning of the *Rhg1* locus in PI 88788 and Peking sources have characterized *GmSNAP18* as a major gene imparting SCN resistance (Cook et al. 2012, 2014; Liu et al. 2017). The characterization of *SNAP* subfamily genes has demonstrated that the *SNAP* genes in soybean undergo co-regulation after SCN infection (Lakhssassi et al. 2017). The detection of *SNAP* genes at resistance loci *rhg1-a*, *rhg1-b*, and *rhg2* highlights the crucial role of *SNAPs* in mediating SCN resistance against multiple virulent SCN populations and more recently reniform nematode (Lakhssassi et al. 2017; Usovsky et al. 2021b).

The epistatic interaction between *rhg1-a* and *Rhg4* governs SCN HG type 0 (Race 3) resistance in Peking-type sources (Meksem et al. 2001; Concibido et al. 2004; Brucker et al. 2005; Liu et al. 2017). However, SCN HG type 0 (Race 3) resistant sources categorized as Peking type (Peking, PI 90763, PI 437654, and PI 89772) display distinct phenotypic responses against other SCN populations (Anand and Shumway 1985; Niblack 2005; Concibido et al. 2004; Niblack et al. 2006) that suggest a role for additional resistance genes. Efforts to determine the differential phenotypic responses within Peking type sources had been previously conducted by genetic analysis using mapping populations derived from complementary crosses between Peking type sources (Thomas et al. 1975; Anand and Sharma 1995). However, an epistatic interaction of the *rhg1-a* and *rhg2* loci conferring resistance to multiple virulent SCN populations had not been reported. Consistent detection of *Rhg1* and *Rhg4* loci with major effects and the detection of other minor effect QTL may have potentially camouflaged the epistatic interaction between these loci. Using a genetic mapping strategy and testing of different SCN resistance loci combinations with multiple virulent SCN populations, we have demonstrated an epistatic interaction between *rhg1-a* and *rhg2* that explains resistance against SCN HG type 2.5.7 (Races 1 and 5) and HG type 1.2.5.7 (Race 2) populations. SCN resistance associated with *rhg2* has been previously detected in PI 89772, PI 438489B, PI 404198A, PI 437654, PI 494182, and PI 84751 (Yue et al. 2001a, b; Guo et al. 2006; Wu et al. 2009; St-Amour et al. 2020; Suzuki et al. 2020); however, none of these reports had identified its distinct role in SCN resistance through an epistatic interaction with the *rhg1-a* locus (Yue et al. 2001a, b; Guo et al. 2005; Wu et al. 2009; Lakhssassi et al. 2017; St-Amour et al. 2020; Suzuki et al. 2020). As such, the *rhg2* gene was inadvertently reported as a minor effect QTL (Lakhssassi et al. 2017; St-Amour et al. 2020; Suzuki et al. 2020).

Emergence and expansion of virulent SCN HG type 2 populations have increased along with excessive utilization of PI 88788-type resistance (*rhg1-b* locus) (Niblack et al. 2008; McCarville et al. 2017; Howland et al. 2018). This breakdown of PI 88788-type resistance resulted due to the selection pressure which facilitated shifts in SCN virulence (Niblack 2005; Niblack et al. 2008; Meinhardt et al. 2021). Hence, there is an urgent necessity for the deployment of alternative sources of resistance apart from PI 88788 to manage yield losses due to SCN (McCarville et al. 2017). SCN resistance loci pyramiding has been proposed as an important strategy for SCN management (Brzostowski and Diers 2017; Yu and Diers 2017; Meinhardt et al. 2021) which requires a solid understanding of the complex interactions among SCN resistance loci from varying soybean germplasm against different SCN populations. Efforts directed at bolstering resistance to SCN populations that have overcome *rhg1-b* by pyramiding with *G. soja* QTL *cqSCN-006*, *cqSCN-007* from PI 468916, and Chr. 10 QTL from PI 567516C SCN resistance have shown some success (Brzostowski and Diers 2017; Yu and Diers 2017; Meinhardt et al. 2021). However, recent studies have demonstrated the potential risks in the generation of more virulent SCN populations by employing SCN resistance combinations without studying the impact on SCN virulence shifts (Chen 2020; Meinhardt et al. 2021). Here, we demonstrate that pyramiding *rhg1-a* and *rhg2* provides an effective strategy for developing SCN resistance to cope with the rapid increase in HG type 2 and HG type 1.2 SCN field populations.

## Summary

Through genetic mapping approaches, we identified a major role for *rhg2* in SCN resistance through a unique epistatic interaction with *rhg1-a* in PI 90763 that provides resistance against multiple virulent SCN populations. The stacking of *rhg1-a* with *rhg2* provides a clear, achievable, and relatively fast solution to diversify commercially available soybean cultivars. It also offers an additional resistance rotation option for sustainable SCN management. Furthermore, the two-gene model breeding strategy recommended here is pragmatic and straightforward, which does not require testing for copy number variation of resistance loci. Thus, we propose the two-gene model as a novel breeding strategy for the next generation of SCN-resistant cultivars.

## Supplementary Information

Below is the link to the electronic supplementary material.

Supplementary file1 (DOCX 24 kb)

Supplementary file2 (DOCX 472 kb)

## Data Availability

The datasets generated during and/or analyzed during the current study are available from the corresponding author on reasonable request.
