# Supplementary material for: Epistatic interaction between Rhg1-a and Rhg2 in PI 90763 confers resistance to virulent soybean cyst nematode populations
Source: Theor Appl Genet. 2022 Apr 5;135(6):2025–39. doi: 10.1007/s00122-022-04091-2 (PMC9205835; doi:10.1007/s00122-022-04091-2)
Supplement: Supplementary file 1 — Supplementary file1 (DOCX 24 kb) [file 122_2022_4091_MOESM1_ESM.docx]

**Supplemental Table 1.** *Heterodera glycines* type (HG type) and race test of SCN population TN22 used in SCN screening of pop1 (SA13-1385 x PI 90763) and pop2 (LD11-2170 x PI 90763).

| **Indicator Line** | **Name** | **FI [%] ^a^** | **HG Type^b^** | **FI [%] ^a^** | **HG Type^b^** |
| --- | --- | --- | --- | --- | --- |
|  |  | pop1 | | pop2 | |
| 1 | Peking | 12 | 1 | 18 | 1 |
| 2 | PI 88788 | 110 | 2 | 99 | 2 |
| 3 | PI 90763 | 0 |  | 0 |  |
| 4 | PI 437654 | 0 |  | 0 |  |
| 5 | PI 209332 | 121 | 5 | 94 | 5 |
| 6 | PI 89772 | 1 |  | 0 |  |
| 7 | Cloud | 105 | 7 | 80 | 7 |
|  | Pickett | 65 |  | 72 |  |
|  | Williams-82 | 100 (189**^c^**) |  | 100 (334**^c^**) |  |

Female indices were calculated using the following formula: FI [%] = [average number of female nematodes on the test line (5 reps) / average number of female nematodes on the susceptible Williams-82] × 100 (Schmitt and Shannon 1992).

^b^ HG type of TN22 was scored when FI was greater than 10 on seven indicator lines.

^c^ Average number of cysts across 5 replications

**Supplemental Table 2.** Quantitative trait loci for resistance to SCN population TN22 (HG type 1.2.5.7) mapped using composite interval mapping in 303 F_3:4_ lines of pop1 (SA13-1385 x PI 90763) and 251 F_3:4_ lines of pop2 (LD11-2170 x PI 90763).

| **Population** | **Peak Marker** | **Peak Position ^a^** | **Confidence Interval Markers** | **Confidence Interval Position ^a^** | **Confidence Interval Size** | **LOD** | **PV ^b^** |
| --- | --- | --- | --- | --- | --- | --- | --- |
| pop1 | Gm11_37408299 | 32959788 | Gm11_37237023 - Gm11_37749863 | 32784991 - 33309696 | 575 Kbp | 41.18 | 62.4 |
|  | Gm18_1909453 | 1909982 | Gm18_1235422 -  Gm18_1957770 | 1235758 - 1958299 | 723 Kbp | 41.89 | 56.6 |
|  | Gm11_37408299 *  Gm18_1909453 | NA | NA | NA | NA | 29.9 | 32.3 |
| pop2 | Gm18_1562162 | 1562536 | Gm18_830106 -Gm18_1909982 | 830106 - 1909982 | 1080 Kbp | 79.3 | 86.7 |

**Supplemental Table 3.** Allelic effect of peak markers associated with detected QTL of resistance against SCN population TN22 (HG type 1.2.5.7) in (A) 303 F_3:4_ lines of pop1 (SA13-1385 X PI 90763), and (B) 251 F_3:4_ lines of pop2 (LD11-2170 X PI 90763).

A.

| **Means** | Gm18_1909453_T_C  (AA) | Gm18_1909453_T_C  (AB) | Gm18_1909453_T_C  (BB) | QTL effect |
| --- | --- | --- | --- | --- |
| Gm11_37408299_G_A (AA) | 119.6 | 124.0 | 127.3 | -107.6 |
| Gm11_37408299_G_A (AB) | 122.3 | 101.7 | 83.7 |  |
| Gm11_37408299_G_A (BB) | 118.9 | 97.8 | 12.0 |  |

B.

| Marker Effects | Gm18_1562162_G_A  (AA) | Gm18_1562162_G_A  (AB) | Gm18_1562162_G_A  (BB) | QTL effect |
| --- | --- | --- | --- | --- |
| Means | 116.7 | 93.9 | 14.9 | -101.8 |

QTL effect

**Supplemental Table 4.** Primer sequences of Kompetitive allele-specific PCR (KASP) assays used in fine-mapping of SCN resistance gene associated with *rhg2* region

|  | **Primer** | **Position** | **R/S Allele** | **Forward_HEX_Allele_X_(WT)** | **Forward_FAM_Allele_Y_(MUT)** | **Reverse_Common** |
| --- | --- | --- | --- | --- | --- | --- |
| 1 | MU-26 | 32786551 | C/A | ACAACTTTGTGATTTATACTAGTAATACATTTT | ACAACTTTGTGATTTATACTAGTAATACATTTG | GTTGTGAAAACATACTCTAGTGACACATTT |
| 2 | MU-28 | 32817860 | C/T | TTCAGTCTATTAAATTTGGCACTATACGT | CAGTCTATTAAATTTGGCACTATACGC | CACTACTAGTGCATAAGAGGCTCCTA |
| 3 | MU-29 | 32826382 | T/C | ATCCGTGGAAAGTGAGTGGTGC | CATCCGTGGAAAGTGAGTGGTGT | CAACTTCCTTCAATGCCGGAGGTAA |
| 4 | MU-30 | 32836489 | A/C | CATATGATACTATGATTTAGGTTTCCATC | ATCATATGATACTATGATTTAGGTTTCCATA | CACAAGGACATAAAAGAACTATGATCGGAT |
| 5 | MU-31 | 32848076 | G/A | GTGAGTTAATTAACCAACTGCTACGT | GTGAGTTAATTAACCAACTGCTACGC | GACACACCAAATCAACGTGGTAACATTTA |
| 6 | MU-32 | 32860666 | C/T | CAATTTACTTACATATTTCATAATAAACCGAA | CAATTTACTTACATATTTCATAATAAACCGAG | GGTGCAACCCAGAAGAATTTGACCAA |
| 7 | MU-33 | 32889293 | A/G | GTCTTTGACAGAACCCAGTTAGC | GTGTCTTTGACAGAACCCAGTTAGT | TTGACTGAAGTGTCACGTGGTGGAA |
| 8 | MU-34 | 32898945 | G/A | GGTTCAATGATTCTTGCCTTCACGA | GTTCAATGATTCTTGCCTTCACGG | CTCTCTACCTACCTTTTCATTATGTTAGTA |
| 9 | MU-16 | 32902214 | T/C | CCAAAATCAAAGATCCAAGTAGTCGG | CCAAAATCAAAGATCCAAGTAGTCGA | ACCCTCTATGTCGCTTCCATCCTT |
| 10 | MU-35 | 32906157 | A/T | ATGGATGAATCAGCACCCGGT | CTATGGATGAATCAGCACCCGGA | TCTTTTCTGGAGATGATTTTGGTGGTGAA |
| 11 | MU-36 | 32915331 | A/T | AATCGCGGTCGCGGCCAATTT | AATCGCGGTCGCGGCCAATTA | GATTTGGGCGGCAACATCAAGGTTT |
| 12 | MU-37 | 32929775 | A/G | ACATGTCAATCCATAAATGTTGCGTAG | AACATGTCAATCCATAAATGTTGCGTAA | ATCTTTTTAAGTTCACGCGACGCTCATAA |
| 13 | MU-38 | 32932038 | T/C | ACACAATCCACAGAGACTGCTTATC | CACACAATCCACAGAGACTGCTTATT | GCTGTTAATGGACTGCAAACTGATTTCTT |
| 14 | MU-39 | 32938436 | G/C | GATTGCATTGGTGGATGGGACTG | GCATTGGTGGATGGGACTC | CAGTCATGGGTCTTGATGCCCTAA |
| 15 | MU-41 | 32955580 | G/A | GTAATGCAAAATATAAGTAATTTGAAACGATGT | AATGCAAAATATAAGTAATTTGAAACGATGC | GTGTCCCTCTCAAACTTTATACATGTTATT |
| 16 | MU-42 | 32964028 | A/G | CAATATATGGATTTTCCTTCTTGTTCAATC | ATACAATATATGGATTTTCCTTCTTGTTCAATT | GTTCCCTGAAAGTGGAGCAAGTTGTA |
| 17 | SNAP11-1 | 32968127 | T/A | AAGATGAACAACTAGACAGACACACAT | AAGATGAACAACTAGACAGACACACAA | GGCGGTTGGACCTATTGTTTGATGAT |
| 18 | MU-44 | 32968423 | G/A | GACTCTCAAACTAATTGAGATAAGTTAGAAA | ACTCTCAAACTAATTGAGATAAGTTAGAAG | GGGGGAGGCAATAGCTTACCTATAA |
| 19 | MU-45 | 32976418 | G/A | CCTAGCATCAAAGATCATTCACCA | CCTAGCATCAAAGATCATTCACCG | GCAATTTTCCCTTTAGATTTGCTCCTCAT |
| 20 | MU-46 | 32988281 | G/A | GAAACAGAATCTTATCAGTTTCTTCAACTTTA | AACAGAATCTTATCAGTTTCTTCAACTTTG | CTTCTTCATGGACTTGGCAGTTGCAA |
| 21 | MU-47 | 32992184 | T/A | ATTTGTATTTATTTGCATCTAATCAAAATAA | CTATTTGTATTTATTTGCATCTAATCAAAATAT | GGTCATCATTCATATCCTATAGTTCAGGTA |
| 22 | MU-48 | 32999963 | C/A | CAAGTATCTGCAGAGGATAAATAATCTCA | AGTATCTGCAGAGGATAAATAATCTCC | GAGTAATTACAAAGGGCAAAAGGGGTAAT |
| 23 | MU-52 | 33075108 | A/G | AAAGTGTTATAGTGTGTAATTATATCCTGC | TAAAGTGTTATAGTGTGTAATTATATCCTGT | CGCAAATATGACAAAATTAATTATGATGTG |
| 24 | MU-54 | 33130991 | G/A | AGTTTCGTAACTTGTGTATCTGTGATAA | GTTTCGTAACTTGTGTATCTGTGATAG | CTAGAACTGCCCAGTGCGAATTGAT |
| 25 | MU-58 | 33237128 | G/T | GTAATTCAAAAAATCTCCAATACTTTTCAGATT | AATTCAAAAAATCTCCAATACTTTTCAGATG | GGATGTGATATTTTTCCGTATCCTACAAAT |
