# Supplementary material for: Epistatic interaction between Rhg1-a and Rhg2 in PI 90763 confers resistance to virulent soybean cyst nematode populations
Source: Theor Appl Genet. 2022 Apr 5;135(6):2025–39. doi: 10.1007/s00122-022-04091-2 (PMC9205835; doi:10.1007/s00122-022-04091-2)
Supplement: Supplementary file 2 — Supplementary file2 (DOCX 472 kb) [file 122_2022_4091_MOESM2_ESM.docx]

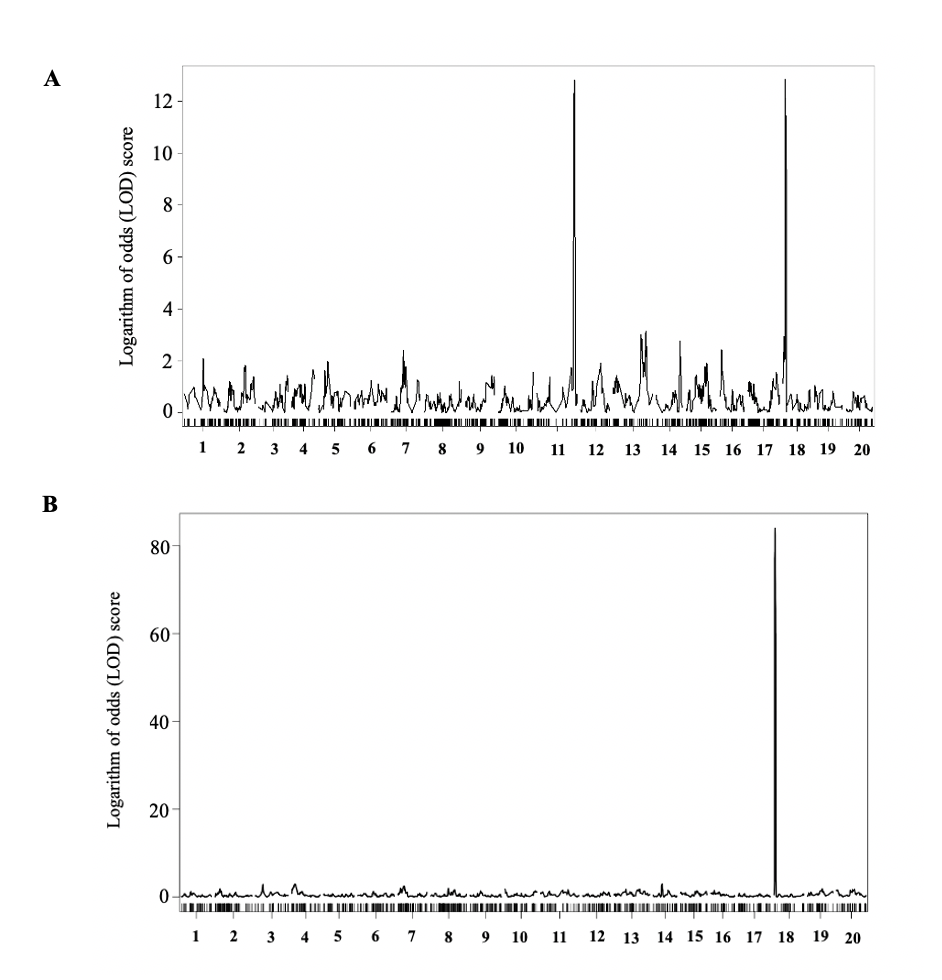


**Supplemental Figure 1.** Quantitative trait loci peaks for resistance to SCN population TN22 (HG type 1.2.5.7 detected for (A) 303 F_3:4_ lines from pop1 (SA13-1385 x PI 90763), and (B) 251 F_3:4_ lines from pop2 (LD11-2170 x PI 90763). The output has been created in Rstudio using composite interval mapping with qtl package as reported by Broman and (Sen 2009). LOD score threshold was estimated from 1000 permutations at α ≤ 0.05 (5.65 for pop1; 18.69 for pop2).

**
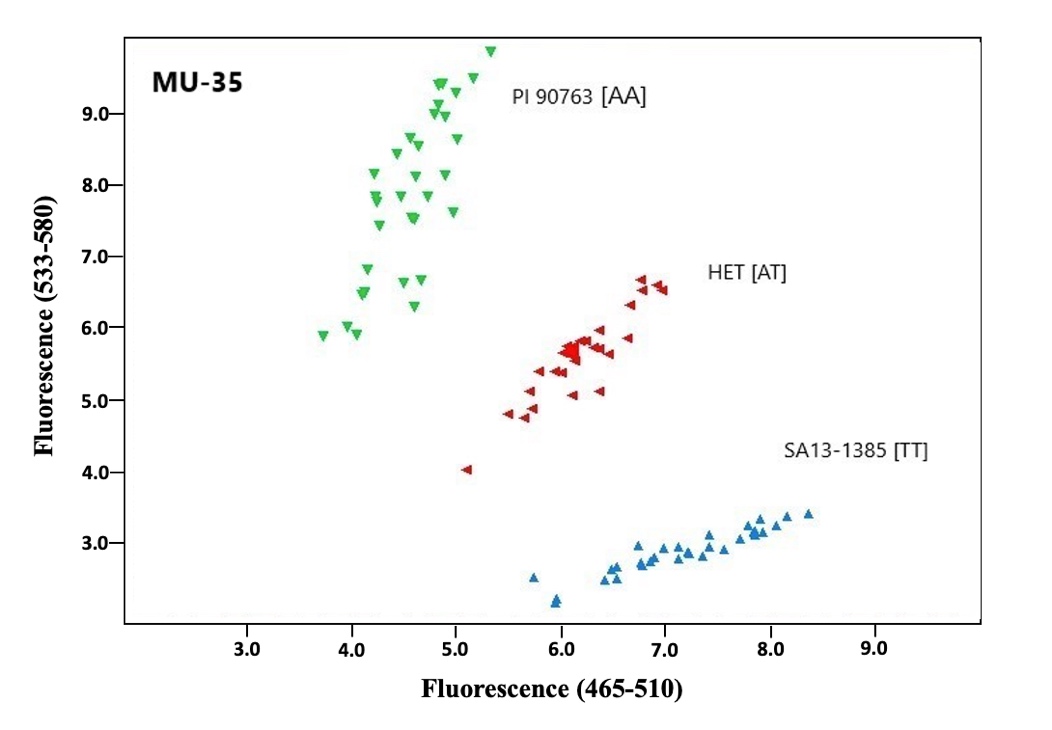
**

**
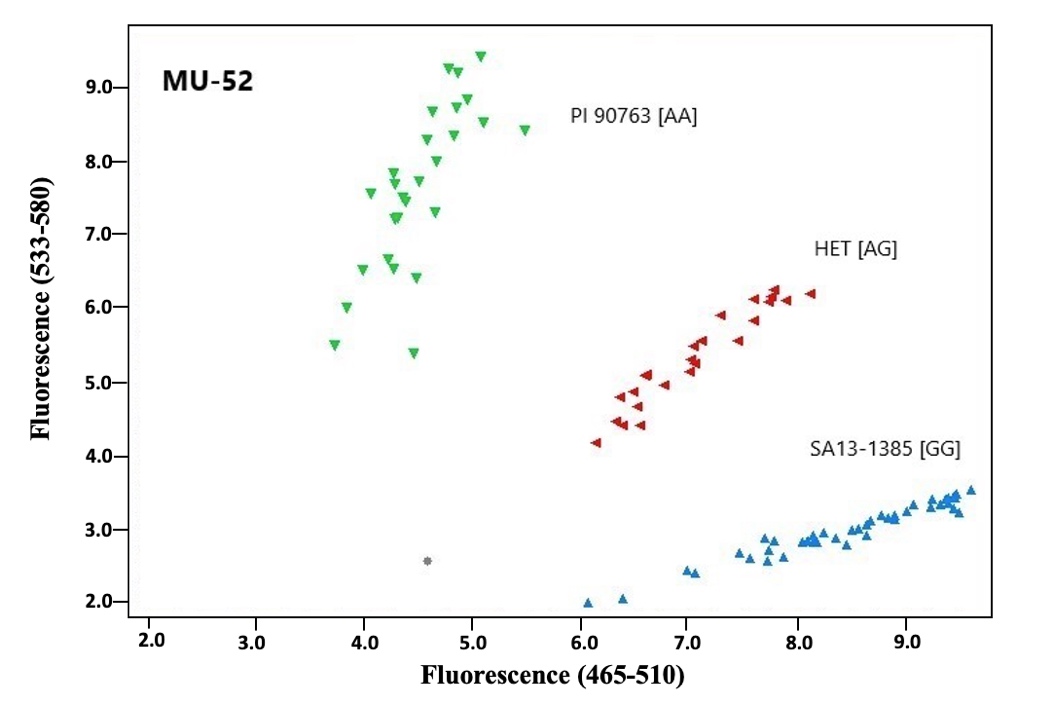
**

**Supplemental Figure** **2.** Endpoint fluorescence scattering plot of the KASP assays flanking *rhg2* region, MU-35, and MU-52, tested in a set of fine-mapping lines. Allele-specific HEX primer (WT) was reported in blue, and allele-specific FAM primer (mutant) was reported in green, heterozygous (HET) lines were marked in red. The X-axis displays fluorescence of FAM at 523 nm to 568 nm, and the Y-axis displays fluorescence of HEX at 483 nm to 533nm.
